# Milk somatic cell DNA isolation and characterization of κ-casein gene in Halari donkey milk

**DOI:** 10.1016/j.heliyon.2024.e24991

**Published:** 2024-01-19

**Authors:** Prashant Singh, Anuradha Bhardwaj, Varij Nayan, Ram Avatar Legha, Yash Pal, Sonali Soni, Shiv Kumar Giri, T.K. Bhattacharya

**Affiliations:** a ICAR-National Research Centre on Equines, Hisar 125001 (Haryana), India; b ICAR- Central Institute for Research on Buffaloes, Hisar 125001 (Haryana), India; c Department of Biotechnology (SBAS), Maharaja Agrasen University, Baddi 174103 (Solan) HP, India

**Keywords:** Halari donkey, Milk, Somatic cells, *CSN3*

## Abstract

Halari donkey breed is one of the indigenous breeds of India and its population is rapidly decreasing. The Jenny milk is more similar to human milk, exhibits a wide range of probiotic diversity and hypo-allergenicity, especially among infants suffering from cow and buffalo milk protein allergy. Some studies indicated low levels of κ-casein fraction of casein protein in donkey milk. The k-casein gene was not amplified from the DNA derived from the milk somatic cells of Halari donkeys. The Halari donkey milk has low somatic cells count. We report the first isolation of DNA from milk somatic cells of Halari donkeys with subsequent characterization of k-casein gene. Through our work, we showed that the milk somatic cells can be used as a non-invasive source for DNA isolation towards molecular studies. It also proved the presence of k-casein gene in Halari donkey milk by its amplification from isolated DNA.

## 1 Introduction

Donkeys (*Equus asinus*) have been used as a common animal in agricultural production and transportation [1], having an important commercial and economic value in meat, skin, and milk production [2]. With unique composition and functional properties, donkey milk has recently gained much interest and is now considered a gold mine for the future [[3], [4], [5], [6]]. It is considered an alternate milk source for infant consumption because of its similarity to human milk [7,8] and may also be considered a new dietetic food for human consumption owing to its benefits [[9], [10], [11], [12]]. Because of its hypoallergenic properties, donkey milk is considered a useful alternative for children affected by cow milk protein allergy [13,14] and by cow milk food protein–induced enterocolitis syndrome, as recently demonstrated in a pilot study [15]. The donkey milk is reported to have less casein fractions and with antioxidant properties [16].

The DNA from mammalian sources has become an important tool which can be used in a vast spectrum of molecular biology applications including the molecular genetic analyses, biotechnological applications, gene editing, food traceability and quality control, animal conservation efforts as well as in the forensic and veterinary-legal applications. In this regard, the milk presents itself as an easily collected, simple, readily-available and non-invasive source of DNA. It is also animal friendly and with minimal concern of animal welfare and consistent with Brambell's five freedom [17,18]. Milk somatic cells include the epithelial cells from the gland and leukocytes from the blood and the milk somatic cell count (MSCC) has become the basis for abnormal milk control programs. The donkey milk has a low somatic cell count suggestive of good mammary gland health [19,20]. The DNA isolation from donkey milk somatic cells is therefore not easy. Here, we demonstrated the DNA isolation from donkey milk somatic cells, and it was further utilized for characterization of κ-casein gene.

Donkey milk casein fraction is represented mainly by αs1-and β-caseins and αs2-casein and k-casein in small amounts [11,21]. The presence of κ-casein in equine milk has been an issue of debate for several years; several authors have reported its absence but recent studies indicate its presence, albeit at a very low level [22]. κ-Casein and αs1-casein, are present in very small quantities in donkey milk which is more similar to the human casein than bovine casein [7]. These proteins have been characterized in other donkey breeds, mainly using blood as a source, but it is yet to be characterized at DNA level using the milk somatic cells in the Halari donkey breed, a native breed of India. , The κ-casein, a variant of casein milk protein, has significant role in the adaptability among infants as κ-casein plays a major role in cow milk protein allergy.

In India, the population of donkeys is rapidly declining [23]. The genetic diversity study was conducted with a panel of 24 polymorphic microsatellites revealing a high number of alleles and heterozygosity in all the Indian donkey clusters available in different agro-climatic regions [24]. However, this important breed*,* Halari donkey, is facing the threat of genetic erosion because of its rapid population degradation. The present work provided for the DNA isolation form Halari donkey milk somatic cells and subsequent elucidation of k-casein presence at DNA level and further characterization. The milk somatic cells were used in this study as they are representative of the mammary gland tissue and provide a non-invasive source of nucleic acid [25]. The results will further help in characterizing other Halari donkey milk proteins.

## 2 Materials and method

### 2.1 Sampling

About 10 mL of donkey milk samples from 10 animals (n = 10) were collected from the Jenny Dairy Unit located at National Research Centre on Equines, Hisar, Haryana, India by hand milking after cleaning the udder with 70 % ethanol. Thus, 10 biological replicates were employed. The Halari lactating jennies were reared in similar housing, feeding (same plane of nutrition) and management conditions. The samples were treated separately. The samples were filtered separately with muslin cloth and then transferred into a new sterile 15 ml centrifuge tube and kept at 4 °C for 24 h till the DNA extraction. The samples were collected as per the guidelines of Institute Animal Ethics Committee.

### 2.2 DNA extraction

DNA extraction from the milk somatic cells was performed as described earlier [26] with certain modifications. Ten mL of whole milk was centrifuged at 400×*g* for 10 min, 4 °C. The supernatant was discarded, leaving some amount behind and the pellet was transferred into a 1.5 mL Eppendorf tube and centrifuged again, and the supernatant was discarded. The pellet was washed with 500 μL wash buffer (15 mM Tris-HCl (pH 7.4–7.6), 25 mM NaCl, 5 mM MgCl_2_,15 mM Na_2_HPO_4_, 2.5 mM EDTA, 1 % sucrose) and centrifuged at 400×*g*, 10 min, 4 °C, and the supernatant was discarded. Washing was performed 3 times. Afterward, the pellet was dissolved in 1 mL lysis buffer (pH 8.8; 6 % SDS, 3 mM MgCl_2_,15 mM Tris-HCl, 0.5 % DMSO, 6 % acetone) and the solution was incubated for 30 min at 65 °C. DNA was precipitated by mixing 200 μL cold isopropanol in the solution and centrifuged at 10000×*g* for 1 min at room temperature. The supernatant was discarded. The DNA pellet was washed with 100 μL 75 % ethanol and it was centrifuged at 10000×*g* for 1 min at RT. It was repeated two times. Pellets were air-dried and suspended in 20 μL nuclease-free water. DNA size and quality were evaluated by electrophoresis in 1.0 % agarose gel (Lonza biosciences) including ethidium bromide. Electrophoresis was carried out in 1 x TAE solution, at 80 V for 45 min and then the gel was observed under a UV light image analyzer (Syngene, G:BOX)

### 2.3 Polymerase chain reaction

PCR was performed specifically to amplify the donkey κ-casein (*CSN3)* gene [27]. A total of 235 bp of the κ-casein gene (*CSN3*) was amplified using the given primers- *F: 5′-GATGACAACTCTATTTCCCCCT-3′ and R: 5′-CCAGGGTCAGGTCTTGCT-3.* The genomic DNA extracted from the milk somatic cells were used as a template in the PCR reaction setup. A total volume of 25 μL of reaction mixture contained 12.5 μL of PCR master mix (Thermo Fisher Scientific), 1 μL of each forward and reverse primer and 2 μL of the DNA sample. The PCR conditions were set as 4 min at 95 ^∘^C, 35 cycles of 95 ^∘^C for 1 min, 57 ^∘^C annealing for 1 min, 72 ^∘^C for 1 min, with a final extension at 72 ^∘^C for 10 min. GAPDH was taken as the control gene.

### 2.4 Gel electrophoresis

The PCR products were analyzed by electrophoresis in 1.5 % agarose gel (Lonza biosciences) stained with ethidium bromide in a 1 x TAE buffer at 80 V for 45 min. A 100 bp DNA ladder was run to assure the size of the amplification product. A 235 bp DNA fragment indicated the presence of the donkey κ-casein gene (*CSN3)*. Sanger sequencing was performed for the amplified PCR products using the forward and reverse primers with the help of M/s AgriGenome Pvt Ltd to ascertain the sequence of amplicon. The PCR provided only a qualitative estimate of the CSN3 gene (its presence or absence) and electrophoresed in agarose gel electrophoresis. The gel was visualized under a UV light image analyzer (Syngene, G:BOX F3; camera: Synoptic 12.8 M; GeneSyn version:1.69) using the GeneSys image capture software v 1.3.1.0. The PCR product sequencing confirmed the gene sequence.

### 2.5 Data analysis

The qualitative PCR indicated the presence of CSN3 gene amplification and didn't require the statistical analysis as required in a quantitative PCR. The experiments were repeated at least three times to check the reproducibility for the CSN3 gene amplification using the DNA extracted for each milk sample and these amounted to three technical replicates for each of the ten biological replicates.

### 2.6 *In silico* analysis of *CSN3* amino acid sequences

Amino acid sequences of *CSN3* protein were retrieved from NCBI GenBank of the following animals: donkey, horse, cattle, buffalo, goat, sheep, yak, camel, llama, mithun, human, and zebra. Multiple sequence alignment of the peptide sequences was done by the MEGA 11.0 program. Further, molecular phylogenetic analysis was done from the data provided by multiple sequence alignment patterns of amino acids following the Maximum-Likelihood method based on 1000 bootstraps replications and Jones-Taylor-Thornton (JTT) matrix-based model by MEGA 11.0 [28].

### 2.7 Statistical analysis of samples

10 number of milk samples (n = 10) were collected aseptically and DNA was isolated from each of them through our protocol with 100 % efficiency. The “safety through independent experimental repeats” approach was adopted. The genomic DNA is routinely isolated from Halari donkey blood samples using the established standard procedures with 100 % efficiency too. The DNA from milk was used for amplification of k-casein gene for the first time in the Halari donkey. The Milk somatic cells were used for the first time for extraction of DNA and subsequent PCR and hence we compared the frequency of positive amplification from milk somatic cells-derived DNA with the gold-standard blood tissue. The positive amplification was coded as 1 and the negative was coded as 0. Results revealed 100 % positive results in both types of samples. The Chi-square test of significance was used to assess the difference in the amplification frequency between milk versus blood-origin DNA PCR efficiency. The results revealed that the efficiencies were similar and there was no statistically significant difference between two assays used (P > 0.99).

## 3 Results

**Fig. 1:**
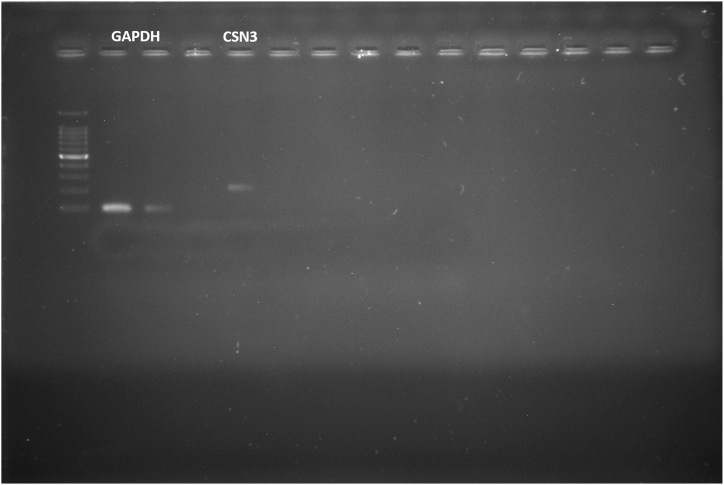
**Gel electrophoresis of PCR products.** 100 bp GAPDH (Lane 2 and 3) control gene and 235 bp donkey-specific CSN3 (Lane 5) gene were amplified indicating the presence and identification of κ-CN gene in the Halari donkey milk.

**Fig. 2:**
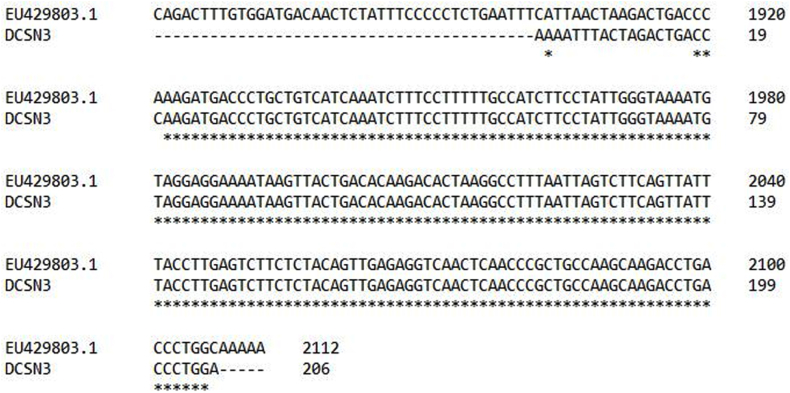
**Clustal W alignment of Sanger Sequence of CSN3 (DCSN3).** The CSN3 gene amplification showed 99 % complementarity with the genomic DNA when BLAST and ClustalW alignment was performed.

**Fig. 3:**
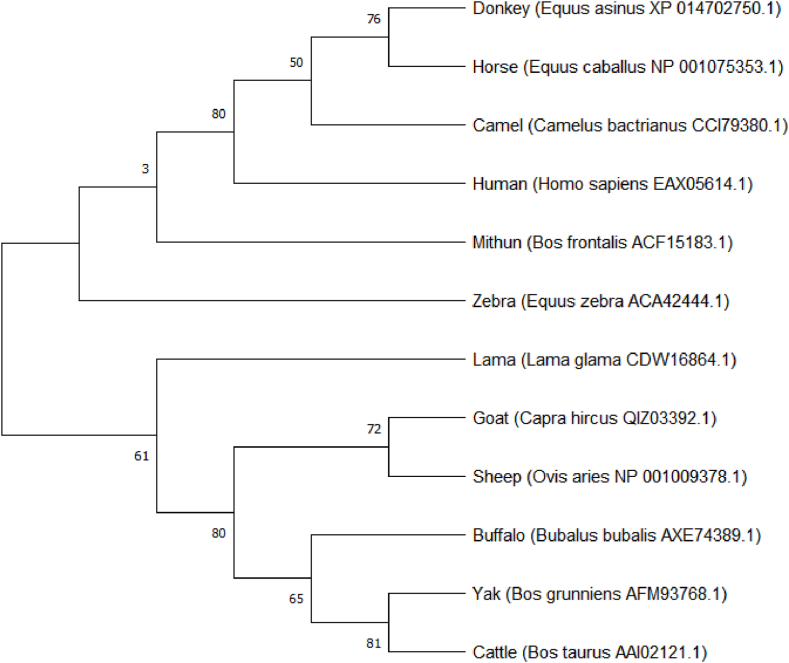
**Phylogenetic relationship of the *CSN3* peptide sequence of Halari donkey (*Equus asinus*) with other mammals.** Molecular phylogenetic analysis was performed from the data provided by the multiple sequence alignment patterns of amino acids. The sources of all the sequence data are represented in the phylogenetic tree. The phylogeny is an un-rooted tree recovered using the maximum likelihood (ML) method based on 1000 bootstrap replications and Jones–Taylor–Thornton (JTT) matrix-based model by the MEGA 11.0 program. Only a bootstrap value greater than 0 % is shown on the branch.

The DNA obtained from the milk somatic cells were run on the gel which showed a clear single band of genomic DNA. No contamination was found as there was no other band (Fig. 1). The PCR amplification products also showed the specific amplification of the *CSN3* gene as there was a single and clear band when the gel was viewed under UV light. No nonspecific primer dimer or product was found. The Sanger sequencing also showed that the sequence amplified was of the required gene which showed 99 % complementarity with the genomic DNA when BLAST and Clustal W alignment was performed (Fig. 2). To identify the phylogenetic relationship of the *CSN3* amino acid sequence of the donkey with other mammalian species, 12 sequences were analyzed: donkey, horse, cattle, buffalo, mithun, camel, llama, goat, sheep, zebra, yak, and human. The result is shown in Fig. 3. The tree indicated greater similarity among donkey, horse, camel human, and zebra as compared to other species.

## 4 Discussion

This is the first study to isolate DNA from the milk somatic cells isolated from the Halari breed of Indian donkeys. The Halari donkey breed of India is recently registered (Accession No: INDIA_DONKEY_0400_HALARI_05002) and is primarily found in the state of Gujarat, India [29]. Our results imply (1) the first report of DNA isolation from the milk of Halari donkey as a non-invasive alternate to other invasive or minimally-invasive methods, (2) the first characterization of k-casein gene from isolated DNA from the Halari donkey milk somatic cells. Studies have suggested the donkey milk importance in human nutrition and since ancient times [[30], [31], [32]] and probable association in metabolic pathways between the different lactation stages [33]. κ-casein is a type of milk protein and is found in very less amounts. However, it plays an important role in its physiological properties. κ caseins, a minor component of the casein micelle surface, provide a hydrophilic layer which is important to the steric stabilization of casein micelles through the inter micellar electrostatic and steric repulsions [34].

It was also reported earlier that female null mouse lacking k-casein gene (Csnk−/−) did not suckle their pups and failed to lactate because of destabilization of the micelles in the lumina of the mammary gland [35]. κ-casein also shows some unique features as compared with the other casein proteins like it is the smallest of the caseins, very lightly phosphorylated, shows low sensitivity to calcium, and is the only casein that may be glycosylated [36]. The study of k-casein was warranted in the Halari donkey milk and the results provided an evidence for it.

No early study has been reported to isolate DNA from the milk somatic cells in donkeys and amplification of the κ-casein gene as per our knowledge. So, it was the first of its successful attempt in our lab. We isolated DNA from 10 milk samples from 10 different animals. The amplification of k-casein gene was done in triplicate for each extracted DNA sample from the milk somatic cells.

## 5 Conclusion

The results of the study led to an efficient method of DNA isolation and characterization of k-casein gene in a non-invasive way. The results correlated with the earlier stated aims and objectives. The casein genes in donkeys were earlier found in association with milk traits in farm animals. Also, the phylogenetic tree shows the greater relation of donkey κ-casein amino acid sequence with humans as compared to cattle and buffalo which indicates its more similar properties with human κ-casein. So, further down-streaming applications of polymerase chain reaction-based amplification of the κ-casein (*CSN3*) gene will advance the case of Halari donkey milk for future research applications and human consumption. Also, the wider adoption of the technique may help towards the threatened donkey population.

## Study Limitations

Currently, there is limited availability and, non-existent legal and social acceptability of donkey milk, alarmingly declining donkey population in India.

## Ethics Declarations

This study was reviewed and approved by Institute Animal Ethics Committee of ICAR-NRCE vide NRCE/CPCSEA/2020-21 dated the January 20, 2021 at S. No. 6.

## Funding Agency

This work was supported by the 10.13039/501100001503Indian Council of Agricultural Research, 10.13039/100021025Ministry of Agriculture and Farmers' Welfare, Govt. of India, who provided financial assistance in the form of an ICAR-NRCE institute grant (IXX15413) and National Livestock Mission, DAHD (MoFAHD) grant (R300121112022-DADF-Dept-Part(l) (E− 22977).

## Data availability

Data associated with this study has been deposited at Mendeley Data with DOI: https://doi.org/10.17632/9w2htf575f.1. (Citation: Singh, Prashant; Bhardwaj, Anuradha; Nayan, Varij; Legha, RA; Pal, Yash; Giri, Shiv Kumar; Bhattacharya, TK (2023), “Files associated with Singh et al., Milk Somatic Cell DNA Isolation and Characterization of k-Casein Gene in Halari Donkey Milk.”, Mendeley Data, V1, doi: 10.17632/9w2htf575f.1)

## CRediT authorship contribution statement

**Prashant Singh:** Writing – original draft, Validation, Investigation, Formal analysis. **Anuradha Bhardwaj:** Writing – review & editing, Writing – original draft, Resources, Project administration, Funding acquisition, Formal analysis, Data curation, Conceptualization. **Varij Nayan:** Writing – review & editing, Writing – original draft, Validation, Resources, Investigation, Formal analysis, Data curation, Conceptualization. **Ram Avatar Legha:** Writing – review & editing, Writing – original draft, Supervision, Resources, Formal analysis. **Yash Pal:** Writing – review & editing, Validation, Supervision, Resources, Formal analysis, Conceptualization. **Sonali Soni:** Writing – original draft, Validation, Software, Methodology, Investigation. **Shiv Kumar Giri:** Writing – review & editing, Visualization, Validation, Investigation, Formal analysis. **T.K. Bhattacharya:** Writing – review & editing, Supervision, Resources, Project administration.

## Declaration of competing interest

The authors declare the following financial interests/personal relationships which may be considered as potential competing interests.
